# 

**Published:** 1900-07

**Authors:** 


### MARRIAGE.

At Woodstown, N. J., June 20th, Dr. Lewis D. Horner to Miss Rachel Lippincott Moore. Dr. Horner graduated at the Veterinary Department, University of Pennsylvania, class of 1898.

### PERSONALS.

Dr. F. M. Perry, formerly of Houlton, Me., has now located at Fort Fairfield.

Dr. Lawrence A. Nolan, of the 1900 graduating class of the Veterinary Department, University of Pennsylvania, has located in his native city, Baltimore, Md., and will shortly open up hospital facilities in connection with his outside practice.

Dr. W. E. A. Wyman, of Milwaukee, has retired from active practice, owing to ill-health, and is now at Burlington, Wis., recuperating his shattered health.

Dr. W. C. Langdon, formerly of the Experiment Station, North Dakota Agricultural College, and who was compelled to resign owing to ill-health, has now located at Omaha, Neb.

Dr. William Kaul, of St. Mary's, Pa., is Secretary of the Driving Association at that point.

Dr. Reginald Wall, of Carbondale, Pa., has just engaged with the British Government to accompany the transportation of horses and mules from New Orleans to South Africa.

As the JOURNAL goes to press with the July number we learn of the serious illness of Dr. Jos. W. Parker, of Kansas City, Kan., Secretary of the Missouri Valley Veterinary Medical Association, from appendicitis. We are not informed as to whether surgical interference was adopted or not.

Dr. J. C. Burnison, of Kenton, Ohio, has just been appointed State Veterinarian and directed to report for duty on August 1st at Wooster Experiment Station. He will give much attention to laboratory and bacteriological work in his new field of labor.

Dr. E. T. Handy, of Eaton Rapids, Mich., has succeeded to the practice of Dr. W. E. A. Wyman at Milwaukee, Wis.

Dr. Robert Ward, of Baltimore, was summoned to direct the destruction of the elephant "Sport," recently hurt by falling from a railroad car while the train was in motion, receiving such serious injuries as to necessitate his destruction. An autopsy was held.

Drs. A. F. Schrieber, Oscar Seeley, E. M. Ranck, and Thos. B. Rayner were Wednesday visitors to the Philadelphia horse-show.

Dr. J. H. Wattles, of Kansas City, Mo., was a visitor to the East in the month of June, stopping at Washington, Baltimore, Philadelphia, and Atlantic City.

Dr. Norris L. Townsend, of East Moorestown, N. J., has recently received an appointment to the Bureau of Animal Industry and assigned for duty to Kansas City, Mo.

Dr. A. R. May, of Boiling Springs, Pa., was a visitor to the "Quaker City" during convention week. He reports professional work in his district especially good.

Dr. Gurdin G. Sill, of Rising Sun, Md., has located in Baltimore as the assistant of Dr. William Dougherty.

Dr. James Beatty, of New York City, has been transferred to the meat-inspection service at Philadelphia, succeeding Dr. W. B E. Miller.

Dr. A. N. Lushington, of Lynchburg, Va., was a visitor to Philadelphia in June, attending the commencement exercises of the Veterinary Department of the University of Pennsylvania.

Dr. J. H. Tennent, of London, Ontario, is a breeder of collie dogs.

Dr. Leonard Pearson, of Philadelphia, addressed the convention of agricultural teachers at the State College, Bellefonte, Pa., early in June.

Dr. A. H. Wallace, of the Bureau of Animal Industry, has been transferred from Chicago to New York City.

Dr. J. P. Foster, of Bangor, South Dakota, reports an outbreak of glanders in that district. He fills the rôle of Deputy State Veterinarian, having graduated from the Ontario Veterinary College, class of 1900.

Dr. H. Young, of Washington, D. C., who graduated in the class of 1900 at the Veterinary Department, University of Pennsylvania, has been appointed assistant veterinarian to the Board of Health, District of Columbia.

Dr. J. C. Foelker, of Allentown, Pa., was a visitor to Philadelphia in June. Dr. Foelker gives very earnest attention to school affairs in his city.

Dr. W. E. Weihe, graduate of the New York State Veterinary College, is now located at West Raleigh, N. C.

Dr. J. C. McNeil, City Veterinarian of Pittsburg, was one of the ushers at the Republican Convention in Philadelphia in June.

Dr. John W. Adams, of the Veterinary Department of the University of Pennsylvania, finds the lot of a medical student in these days a hard one, and sighs for the return of the good old days when many hours of studentship could be whiled away in football sport.

Dr. S. B. Nelson, of Pullman, Washington, addressed the State Medical Society Convention on May 8th on "The Importance of the Relation of Tuberculosis in Domesticated Animals to that of Man."

Dr. Clarence J. Marshall, of Philadelphia, and Dr. Charles F. Oat, of West Chester, Pa., have been selected as veterinarians to the Devon horseshow.

Among the veterinarians noted at the Philadelphia horseshow were Drs. S. J. J. Harger, Robert Gladfelter, W. Horace Hoskins, Alex. Glass, and a number of students of the Veterinary Department of the University of Pennsylvania.

Dr. Harry Walter, of Trenton, N. J., is now the owner and director of a locomobile.

State Veterinarian Meisner, of Maryland, was a visitor to Philadelphia in June, selecting horses at the horseshow sales held in the "Quaker City."

Drs. Olof Schwarzkopf, of Flushing, L. I., and D. B. Fitzpatrick, of Philadelphia, were recently applicants before the army examination board for the positions of army veterinarians.

The death of Dr. A. H. Vose, of Brockton, Mass., is reported to the JOURNAL.

Dr. E. L. Cornman, of the graduating class of 1900 of the Veterinary Department, University of Pennsylvania, has received the appointment of house surgeon to the Veterinary Hospital, succeeding Dr. M. Jacob. Those who seem best able to judge say there will be an unusual filling up of the canine and feline department incidental thereto, owing to the new house-surgeon's popularity among the ladies.

Dr. A. W. Clement and F. H. Mackie, of Baltimore, Md., were summoned to hold an autopsy on the elephant "Jolly" that dropped dead in Baltimore while being exhibited in conjunction with the Elks' convention.

President R. R. Bell, of the New York State Veterinary Society, is putting forth special efforts for a successful meeting at Ithaca, N. Y., in September.

Dr. John P. Miller, formerly of Lebanon, Pa., has located at Reading, Pa., where he has opened an office and hospital.

Dr. R. S. Huidekoper, of Washington, D. C., was an Atlantic City visitor during the early days in July, and found a deal of pleasure in the splendid surf-bathing afforded by this wonderful seashore resort.

Dr. James B. Rayner, of West Chester, Pa., has been on the sick-list for a number of months. More than fifty years of professional work stands to his credit and good name.

Frank P. Wolken, of the Jersey Farm Dairy Company, of St. Louis, Mo., has become a veterinary student, that the company may have the advantages of constant veterinary supervision.

Dr. William Andress, of the graduating class of 1900 of the Veterinary Department, University of Pennsylvania, will take charge of the practice of Dr. Alex. Glass while he sails the deep waters of the Chesapeake in his new launch.

Drs. James J. Joy, of Detroit, Mich., and W. F. Walsh, of New York City, strongly disapprove of the use of acids in treating punctured wounds of the feet, in the June issue of the *Horseshoers' Journal.*

President Pearson and Dr. Huidekoper interviewed President McKinley in June on the need of recognition with rank of the army veterinarian.

Dr. M. H. Reynolds, of St. Anthony Park, Minn., addressed the State Medical Society on June 28th, at Duluth, on "State Work with Infectious Diseases of Domestic Animals."

Dr. J. Boomer, M.D.V., formerly associated with McKillip Veterinary College of Chicago, has gone to San Francisco, Cal.

Dr. P. I. Kershner, formerly of Kansas City, Mo., has located at Manhattan, Kan.

Dr. Daniel Lemay, of the Seventh United States Cavalry, Columbia Barracks, Havana, Cuba, has gone to Bord-a-Plouffe, Montreal Canada, on a four months' leave of absence.

Dr. George P. Harlan, of Fremont, O., is actively interested in building and loan association societies.

